# Neuronal replacement: Concepts, achievements, and call for caution

**DOI:** 10.1016/j.conb.2021.03.014

**Published:** 2021-08

**Authors:** Magdalena Götz, Riccardo Bocchi

**Affiliations:** 1 Physiological Genomics, Biomedical Center (BMC), Ludwig-Maximilians-Universitaet (LMU), Großhaderner Str. 9, 82152 Planegg/Martinsried, Germany; 2 Helmholtz Center Munich, Biomedical Center (BMC), Institute of Stem Cell Research, Großhaderner Str. 9, 82152 Planegg/Martinsried, Germany; 3 SyNergy Excellence Cluster, Munich, Germany

**Keywords:** Neuronal replacement therapies, Transplantation and direct neuronal reprogramming

## Abstract

Regenerative approaches have made such a great progress, now aiming toward replacing the exact neurons lost upon injury or neurodegeneration. Transplantation and direct reprogramming approaches benefit from identification of molecular programs for neuronal subtype specification, allowing engineering of more precise neuronal subtypes. Disentangling subtype diversity from dynamic transcriptional states presents a challenge now. Adequate identity and connectivity is a prerequisite to restore neuronal network function, which is achieved by transplanted neurons generating the correct output and input, depending on the location and injury condition. Direct neuronal reprogramming of local glial cells has also made great progress in achieving high efficiency of conversion, with adequate output connectivity now aiming toward the goal of replacing neurons in a noninvasive approach.

## Highlights

• Restorative medicine aims to replace adequate, specific neuronal subtypes.
• Criteria defining neuronal subtypes, given their high molecular diversity, are needed.
• Single-cell RNA sequencing allows monitoring and refining transplanted neuron subtype identity.
• Direct neuronal reprogramming from glia *in vivo* occurs with high efficiency.
• Criteria for fate conversion versus endogenous neuron gene activation are needed.

## Introduction: concepts and criteria for neuronal replacement

**Fig. 1:**
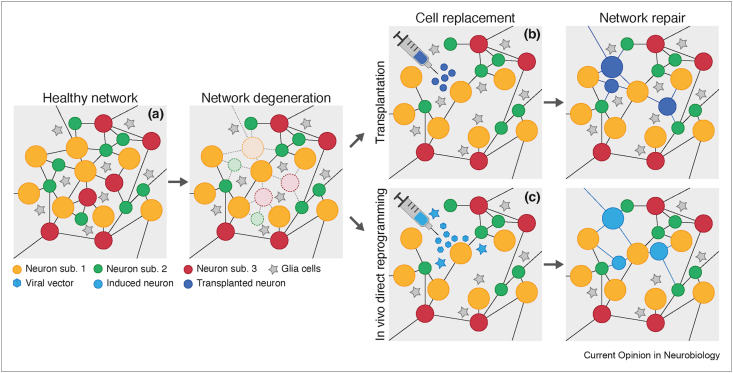
**Neuronal replacement therapies.(a)** Neuronal loss and network degeneration are major hallmarks of both acute brain injuries and neurodegenerative disorders. Regenerative approaches have made great progress, aiming nowadays toward replacing the exact lost neurons and restoring the correct network. Neuronal replacement therapies have mainly focused their efforts on two promising approaches: **(b)** cell transplantation takes advantage of different types of neuronal progenitors as sources of donor cells and **(c)** direct reprogramming of *in loco* glial cells to a neuronal fate by introducing proneural factors via viral vectors.

Loss of neurons is at the core of cognitive and functional failures in various neurological conditions spanning from acute injuries, such as traumatic brain injury and stroke, to a multitude of neurodegenerative diseases, such as Alzheimer disease, Huntington disease, and Parkinson disease (PD). Pioneering transplantation approaches in patients with PD showed an amelioration of symptoms by ectopic transplants of dopaminergic neurons into the basal ganglia, the target region of the lost neurons in the substantia nigra pars compacta (SNpc) [1]. The target site of SNpc dopaminergic neurons (i.e. basal ganglia) was chosen owing to the uncertainty that axons of transplanted neurons would have grown properly in an adult brain. Now we know that young neurons can readily extend axons in an adult brain and even find their correct target regions [2, 3, 4, 5] — a crucial and promising prerequisite for achieving adequate functional repair of neural network activity. This and the knowledge gained in the last decades on transplanted neuron differentiation and integration in adult brains set the stage toward successful neuronal replacement therapies, by replacing the lost neuronal subtypes at their appropriate sites. This would encompass not only the generation of the exact lost neuronal subtype (either from exogenous or endogenous sources) but also the appropriate integration into the pre-existing network, including correct input and output connectivity (Figure 1).

**Fig. 2:**
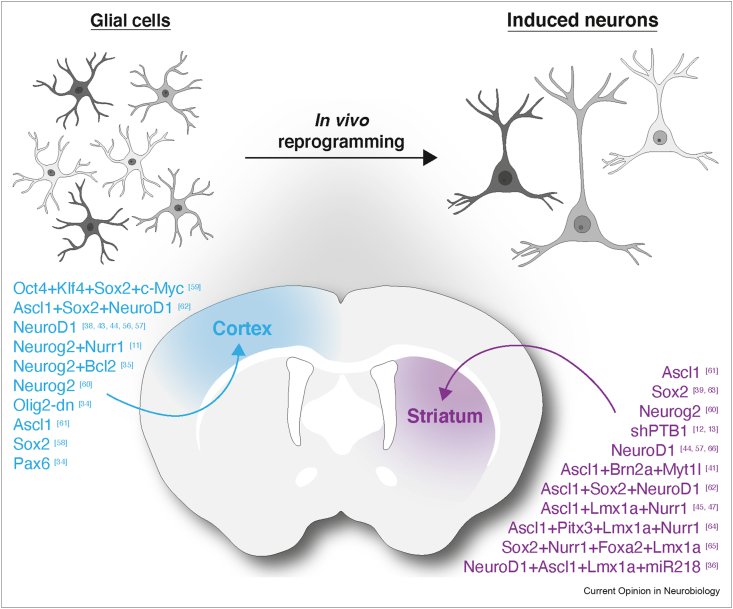
**Combinations of neurogenic factors used for *in vivo* reprogramming in the neocortex and striatum.***In vivo* direct reprogramming of glial cells can be achieved using different cocktails of factors promoting fate conversion into neurons [11∗∗, 12∗∗, 13∗∗,^35^,^36^,^38^,^39^,^41^,^43^, ^44^, ^45^,^47^,^56^, ^57^, ^58^, ^59^, ^60^, ^61^, ^62^, ^63^, ^64^, ^65^, ^66^].

**Fig. 3:**
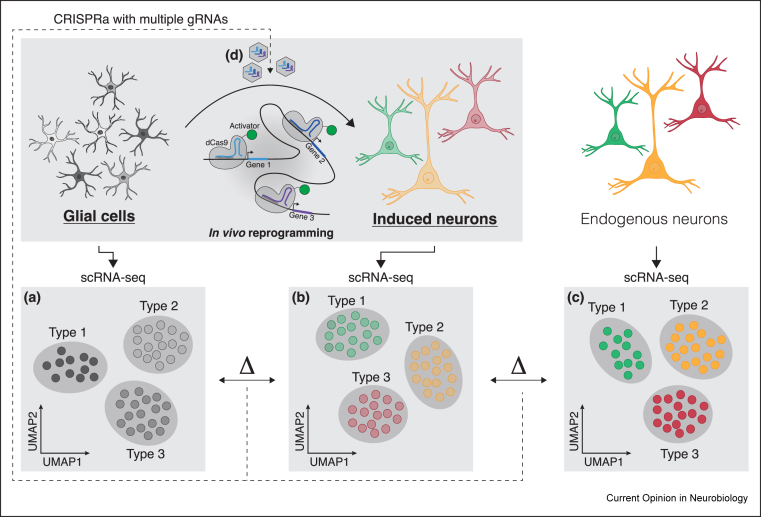
**Single-cell RNA-sequencing (scRNA-seq) and CRISPR-mediated gene activation (CRISPRa) technologies can improve *in vivo* reprogramming.** With scRNA-seq, we can now examine the patterns of gene expression in glial cells **(a)**, induced neurons **(b)**, and endogenous neurons **(c)** at the single-cell level. The comparison of these data sets could ultimately highlight the differences between induced and endogenous neurons, improving the accuracy of reprogramming. This could be achieved by multiplexing viral-transduced gRNAs for selected genes, using CRISPRa **(d)**.

This grand aim also prompts grand questions — first of all, how many neuronal subtypes exist in a given brain region and how many do we need to replace to restore the function of that region. The first question has recently been boosted by advances at the single-cell level that have revealed a plethora of neuronal subtypes, defined by their molecular, electrophysiology, morphology, and connectivity identities [6, 7, 8]. However, the results of brain-wide connectivity studies are still incomplete, especially at single-neuron resolution and for humans and even more so for diseased brains. The latter is highly relevant for a successful repair as symptoms often appear only after a significant loss of neurons, for example, in PD when 70% of the dopaminergic neurons are lost in the SNpc [9,10]. This resilience implies a high degree of plasticity in some neuronal networks, prompting the question of how many neurons need to be replaced for repair of neural network function. The second main question is how newly transplanted neurons integrate into such an altered circuitry, especially given the recent data showing that the input connectome highly depends on the transplantation site and injury condition, which we review in the following section. Importantly, achieving the correct connectivity is still a major challenge for neurons obtained by direct reprogramming *in vivo*, but recent progress has been made on the output axonal projections of induced neurons [11∗∗, 12∗∗, 13∗∗]. Moreover, neurons reprogrammed from local glial cells in the forebrain can now be instructed with great efficiency (Figure 2). The fidelity of the induced neurons is subject to further improvement by comparing single-cell RNA sequencing (scRNA-seq) data from endogenous and induced neurons. This allows correcting the differential gene expression for a more efficient reprogramming, by multiplexing gRNAs using CRISPR-mediated gene activation (Clustered Regularly Interspaced Short Palindromic Repeats, Figure 3) [14]. It is therefore timely to review the achievements and challenges of the major approaches of neuronal replacement therapy — the use of exogenous cells, such as transplantation of neuronal progenitors (including those of human origin), and the more recent approach of direct conversion of non-neuronal cells to neurons.

## Transplantation approaches: generating specific neuronal subtypes that integrate adequately into the pre-existing circuits

To achieve best results for repair, the correct neuronal subtypes have to be regenerated. Embryonic progenitors of a certain brain region were most efficacious in generating the correct neuron subtypes of that region and manage to generate neurons in the adult brain environment that is rather gliogenic [15], while other cells such as adult neural stem cells convert into glial cells when transplanted into the adult brain [1,16,17]. Thus, fetal neuroblasts are role models for cells able to differentiate into neurons upon transplantation into the adult brain parenchyma and hence serve as a blueprint to better understand and implement the mechanisms underlying fate specification. Accordingly, their molecular specification can be used to instruct the differentiation of induced pluripotent stem cells (iPSCs) to the desired neuronal subtype. For example, La Manno et al [18] used scRNA-seq of murine and human fetal ventral midbrain cells to better understand and refine transcriptional networks specifying dopaminergic neurons from this region [19,20]. This matters for the success of transplantation therapies: the better the match to the SNpc type of dopaminergic neurons, the better the reported functional recovery, at least in the mouse models that are used as preclinical readouts [21, 22, 23∗]. This prompts the key question of how many neuron subtypes exist and are lost in the considered human brain region and whether some are more critical to achieve the best functional repair. This question is at an exciting state nowadays, as single-cell omics techniques allow probing for virtually all neuronal subtypes in adult and fetal human brains. For example, human fetal ventral midbrain regions comprise various neuronal clusters, including different developmental stages and maturity identities [24]. It is thus not yet known if there are also several subtypes of dopaminergic neurons within the SNpc region, and if so, it is not known whether these have different functional relevance. However, comparison of scRNA-seq data of fetal cells and iPSC differentiation protocols clearly allows fine-tuning and optimization to gain specificity in the transplanted neuronal subtype [25].

Therefore, the discovery of a plethora of neuronal subtypes in the first wave of brain scRNA-seq prompts the question of their functional relevance. Are the clusters of cells sharing similar gene expression patterns functionally distinct types of neurons, or do they reflect different states of a same subtype (e.g. firing versus not firing neurons or different metabolic states)? If they were molecularly distinct subtypes of neurons, to what extent does each of them matter for function and hence for repair? For example, in the cerebral cortex, a large number of neuronal subtypes have been suggested from pioneering scRNA-seq analyses [7,26], while more recent analysis combining single-cell patching (i.e. electrophysiological properties), filling to examine morphology, and RNA sequencing came to the conclusion that only 3 major subtypes of projection neurons are discernible [6] — even fewer than subtypes classically associated with different cortical layers [27]. This may imply that the more classical parameters, such as neurotransmitter subtype and proper firing patterns, combined with the adequate input connectivity and axonal projection to connect with the correct target may be most relevant for successful repair. Notwithstanding, scRNA-seq provides a unique opportunity to identify the exact transcriptional state of the transplanted neurons [25], in comparison with the endogenous neurons, that are aimed at being replaced.

On a broader level, regenerative neuroscience meets neural network analyses to improve repair strategies with the challenge to integrate new neurons into pre-existing circuits that normally do not integrate new neurons — notably different from development or sites of ongoing adult neurogenesis [28]. The ability to monitor the brain-wide input connectome of a given neuron, owing to development of viral synaptic tracing methods [29], has provided revolutionary and surprising insights into crucial aspects of neuronal functions. Neurons transplanted into the visual cortex upon selective ablation of upper layer neurons could indeed receive the adequate brain-wide input connectome [3,5]. This occurred with precise topographic arrangement and appropriate quantitative differences, with higher innervation density from specific brain regions, closely reflecting the input connectome of the endogenous neurons from this region [3]. Likewise, human iPSC-derived neurons transplanted into a stroke model receive appropriate input connectome despite their rather immature state [23]. This seems to be very different when murine neurons were transplanted into the aging brain or into models of amyloidosis, a hallmark of Alzheimer disease, with highly exuberant local inputs observed in this condition (Thomas, Conzelmann, Grade, Götz, unpublished). Thus, homotopic transplants profoundly differ in their input connectome depending on the host environment, which represents therefore a crucial parameter to bear in mind for therapeutic approaches that in most cases will take place in the aged brain environment.

Interestingly, the input connectome to transplanted ventral midbrain dopaminergic fetal cells has been compared in the homotopic location of ventral midbrain and heterotopically in the striatum [30,31]. This comparison showed that transplanted neurons receive the innervation present in the respective region — input from striatal inhibitory neurons and striatal afferents when transplants were placed in the striatum [30], while receiving ventral midbrain neuron inputs when transplants were placed there [31]. The most striking finding was the extensive input connectome that young and immature neurons received already at 6 weeks after transplantation, a stage at which hardly any axons have yet reached the target region of the striatum [31]. These data also suggest caution about the rabies virus tracing and call for verification by physiological techniques. Taken at face value, afferents seem able to connect to any type of available neurons, at least young transplanted and hence easily excitable neurons. This is good news for replacement therapies, but caution needs to be exerted with regard to whether more fine-tuned intraregional specificity, such as layer-specific connections within the cerebral cortex, could be achieved. Moreover, most of these observations have been probed in experimental conditions of acute neuronal ablation (also in PD models, most of the studies were conducted on acute ablation of SNpc dopaminergic neurons), while the actual chronic disease environment may lead to very different pre-existing connectivity and provide a very different environment for the integration of new neurons, as mentioned previously.

Taken together, replacement therapy via transplantation has entered a new age of scrutiny, allowing the optimization of neuronal subtype specification at an unprecedented fine-tuned level with regard to both gene expression and brain-wide connectome analyses. Excitingly, human cell transplantations into preclinical models allow probing at least some of these aspects of *in vivo* connectivity also for human cells [32].

## Direct neuronal reprogramming: high efficiency conversion and call for further controls

The exciting progress in our knowledge of subtype-specific gene expression and connectivity likewise applies to the neuronal replacement approach that takes advantage of endogenous non-neuronal sources, by direct conversion of local glial cells into induced neurons [33]. Indeed, also the quality of induced neurons has made a breakthrough from initially obtaining very few immature neurons to now achieving fully mature neurons with long axonal projections to their correct target sites [11,34, 35, 36, 37, 38, 39]. It appears that we have even reached the stunning stage of requiring only a single-factor knockdown, to achieve the conversion of astrocytes into the correct neuronal subtype in the respective region [12,13]. These amazing results prompt caution for conceptual and experimental reasons. Conceptually, the apparent ease of reprogramming raises the important issue of how cell fate is normally maintained in an adult organ. If cells can be so readily converted by knocking down a single factor (PTBP1 in this case), how is the physiological downregulation of this factor avoided (or at least controlled), to not have spontaneous cell conversion? Indeed, research on fate maintenance has been boosted by the identification of reprogramming hurdles [40]. At the experimental level, the use of novel viral vector tools requires caution to ensure real reprogramming of glial cells *versus* overexpression/downregulation of the selected factors in endogenous neurons.

The first *in vivo* reprogramming into neurons was achieved by targeting the proliferating glial cells after acute brain injury, using retroviral vectors that selectively integrated their genome only in dividing cells to express the proneural factors such as Pax6, Neurogenin 2, NeuroD1, or Ascl1 (Figure 2) [34,38]. This clearly targeted non-neuronal cells, as neurons do not divide, and reported an efficiency of up to 90% by combining Neurogenin 2 and Bcl-2 [35]. Intriguingly, young induced neurons die preferentially by ferroptosis [35], a cell death mediated by increased lipid peroxidation, which is caused by late or missing conversion of the mitochondrial proteome [14]. Other viral vectors, lentiviral or adeno-associated viral (AAV) vectors, can integrate their genome also in postmitotic cells or remain episomal, respectively. Therefore, transduction depends either on the cells that they infect or on the use of specific promoters. AAV vectors seem best suited as they have high infectivity and the advantage of not integrating into the host cell genome, eliciting a much less inflammatory and reactive gliosis response [11,41]. However, the AAV2/5 vectors used for reprogramming infect dividing and nondividing cells, with a preference for neurons [42]. Glial promoters driving the expression of the Cre recombinase in oligodendrocyte progenitors, astrocytes, or microglia have therefore been used to target expression of the reporter gene and the neurogenic factors to each of these glial cell types, respectively [11∗∗, 12∗∗, 13∗∗,^43^, ^44^, ^45^, ^46^, ^47^]. However, this required very stringent controls to ensure that expression is not aberrantly activated in endogenous neurons, as recently reported to happen in the case of the expression of NeuroD1 under GFAP promoter [48]. So far, only one study has labeled virtually all endogenous neurons before reprogramming, to reveal that most of the induced neurons originated from nonlabeled endogenous non-neuronal cells, namely, astrocytes [11].

To resume with the aim to replace SNpc dopaminergic neurons discussed previously in regard to transplantation, recent publications claimed for astonishing reprogramming of local astrocytes to dopaminergic neurons by knocking down a single factor, namely, PTBP1 [12,13]. In this case, the authors modeled PD loss of dopaminergic neurons in this region by injecting 6-hydroxydopamine, which induces the selective loss of the vast majority of this neuronal subtype [49]. However, other neuronal subtypes such as neighboring interneurons or projection neurons could still be present in the substantia nigra and therefore be infected. Those potentially nonspecifically targeted cells could turn on the expression of the reprogramming constructs and hence change their gene expression. Keeping this in mind, even the elegant experiment of functional silencing of the induced neurons resulting in behavioral impairment [12], does not help clarify whether the neurons responsible of the behavioral ameliorations were silenced endogenous neurons or truly induced neurons. Therefore, it is essential to label endogenous neurons of all types before reprogramming protocol, to distinguish pre-existing neurons from induced neurons. Ideally, this could be combined with genetic fate mapping of the cell types of origin of induced neurons, for example, oligodendrocyte progenitors, astrocytes, or microglia. These controls should help understand if *in vivo* conversion of glial cells into specific dopaminergic neuronal subtypes is indeed so readily possible with just one factor [12,13] or rather requires indeed several factors to be activated [36,47]. Even in the case that more factors are needed, CRISPR-mediated gene activation now allows multiplexing by the use of several gRNAs to activate simultaneously several factors [50]. Using CRISPR technology, Zhou et al [13] reported the intriguing finding that the knockdown of PTBP1 would elicit the conversion into different neuron subtypes as per the targeted CNS regions. Likewise, differences in neuronal subtypes emerging from astrocytes were also observed for different cortical layers [11] and between the cortex and midbrain [12,13] — not only highlighting the importance of astrocyte heterogeneity between regions but also reassuring the aforementioned raised concerns about artefactual labeling of endogenous neurons [51].

New technologies can now further provide insights into the heterogeneity of cells converting into neurons and the mechanisms underlying this conversion. At the same time, they aim at tackling the control issue, to ensure a real reprogramming rather than activating gene expression in endogenous neurons. For example, scRNA-seq combined with lineage tracing [52] would probe the cell of origin, while simultaneously monitoring the transcriptional process of fate conversion and the identity of the emerging neurons. Likewise, chronic *in vivo* imaging should allow watching the conversion of a glial cell into a neuron live and thereby unequivocally identifying the reprogramming process. Indeed, this has been carried out for transplanted neurons uncovering key principles of their differentiation, dendrite pruning and synaptogenesis [3,53].

In regard to axonal connectivity, direct reprogramming has also made great achievements as axons projecting to the correct target regions have been detected from induced neurons [11∗∗, 12∗∗, 13∗∗]. Even more importantly, retrograde tracing from the target region initially labels no converting neurons, but at several months into the conversion process, the induced neurons can be back-traced from their target region [11,12]. This not only is exciting evidence for correct axonal navigation by induced neurons but also strongly supports the idea that these neurons emerge from reprogrammed astrocytes rather than from endogenous neurons.

Therefore, this is an exciting time for the alternative approach to replace lost neurons from endogenous sources, especially also as increasing numbers of reprogramming protocols of human cells have been developed *in vitro* (see, e.g. the study by Nolbrant et al. [37]), and promising efficient conversion has been instructed also *in vivo*.

## Conclusions and perspectives

Both these approaches of neuronal replacement for brain repair pave the way to a future with causative treatments of neuronal loss conditions.

The transplantation approach has reached the clinics, and several clinical trials are ongoing. It should be soon extended to further neurological disease applications, such as stroke [22,23], taking into account the recent results discussed previously about the effect of the environment on network integration. Moreover, iPSC technology encompasses the perspective for autologous transplants, as recently shown for treatment of a patient with PD [32].

Regarding reprogramming of endogenous glial cells, the conversion into neurons seems to occur with high efficiency *in vivo*. Despite the caveats described in the previous section, AAV vectors represent one of the safest vectors for clinical use in patients as they remain episomal and have a low immunogenicity. Thus, using AAV vectors for neuronal reprogramming offers a translational opportunity, especially because some serotypes, such as AAV9, can be systemically delivered and still be able to selectively target glial cells in the central nervous system [54,55]. All these advantages set up neuronal reprogramming as a promising strategy to pursue in the future, to ultimately treat neuronal loss. However, caution is essential not to prematurely excite the hopes of patients.

## Conflict of interest statement

Nothing declared.
